# The Rarest of the Rare: A Case of Primary Cardiac Osteosarcoma With a Review of the Literature

**DOI:** 10.7759/cureus.16492

**Published:** 2021-07-19

**Authors:** Hemendra Mhadgut, Sukesh Manthri, Bahaaeldin Youssef, Devapiran Jaishankar

**Affiliations:** 1 Medical Oncology, East Tennessee State University, Johnson City, USA; 2 Pathology, East Tennessee State University, Johnson City, USA

**Keywords:** osteosarcoma, cardiac tumors, sarcomas, systemic chemotherapy, resection

## Abstract

A 54-year-old female presented with shortness of breath and cyanosis. Work up with chest X-ray and subsequent echocardiogram revealed an intracardiac bi-atrial mass leading to emergent cardiothoracic resection. Pathology was consistent with a primary cardiac high-grade osteosarcoma. Post-resection staging positron emission tomography-computed tomography (PET-CT) showed hypermetabolic mixed lytic and sclerotic lesion of T10 concerning for metastasis. She received five cycles of adriamycin and ifosfamide chemotherapy before discontinuation due to systolic dysfunction. Nine months later, she developed a high tumor burden with progressive disease and was treated with second-line gemcitabine/docetaxel with disappointing results. She is currently on treatment with cyclophosphamide and topotecan as third-line treatment with an excellent clinico-radiographic response. Osteosarcomas are aggressive with a high incidence of recurrence and metastasis. Fewer than 50 cases of primary cardiac osteosarcomas have been reported in the literature. Even though complete resection can be achieved in some cases, long-term results are usually poor. No standard therapy has been established.

## Introduction

Metastatic tumors of the heart are more common than primary tumors. The frequency of primary cardiac tumors is 0.001%-0.03% in an autopsy series [1]. About 75% of these primary cardiac tumors are benign tumors such as myxoma, lipoma, papillary fibroelastoma, and rhabdomyoma [2]. The most frequently reported malignant primary cardiac tumors are sarcomas. Angiosarcomas and myxofibrosarcomas are the most common sarcomas of the heart [3]. Cardiac osteosarcoma, however, is extremely rare. We present a rare case of bi-atrial high-grade osteosarcoma [4]. This case was previously presented as a meeting abstract at the Appalachian Student Research Forum in April, 2020.

## Case presentation

**Figure 1:**
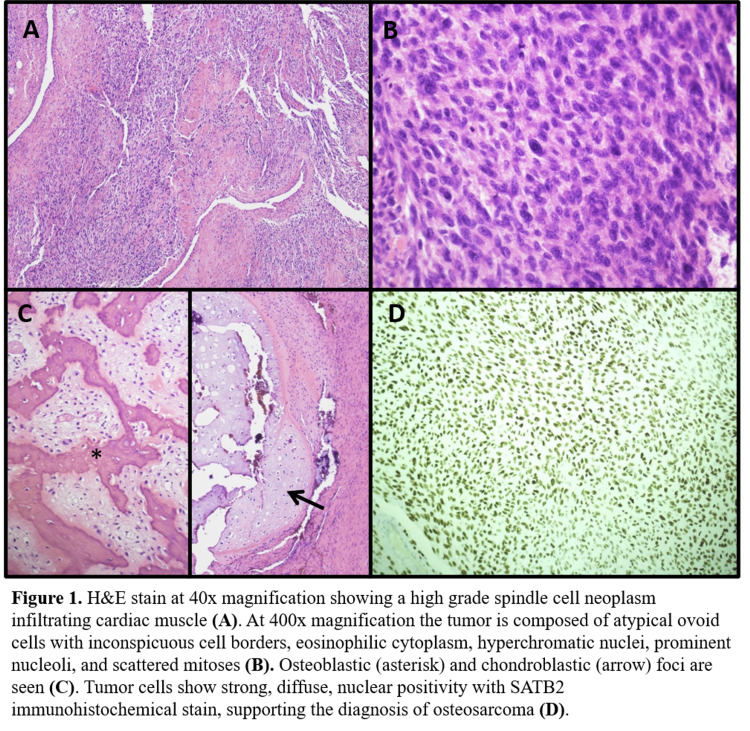
Pathology description

A 54-year-old Hispanic female presented with shortness of breath and was cyanotic on examination while visiting Mexico. Work up included an abnormal chest X-ray, echocardiogram concerning for bi-atrial myoma leading to a cardiothoracic surgical referral. She underwent bi-atrial intracardiac tumor resection in Mexico. Several months prior to her resection, she noted numbness on the side of the face evaluated by her physicians in the United States with a brain MRI and carotid ultrasound/Doppler that was unrevealing. She also remembered an episode of uncontrolled hypertension two years prior to surgery requiring admission to a local hospital in East Tennessee with a cardiology evaluation. Surgical pathology showed extensive undifferentiated spindle cell proliferation with multifocal osteoid production and foci of osseocartilaginous differentiation (Figure 1). There were prominent necrosis and a moderately high mitotic rate (10-19/HPF). Tumor cells were positive for SatB2 and negative for vascular, muscular, or neural markers. This is consistent with a primary cardiac high-grade osteosarcoma. These occur very rarely, usually in the atria, and behave aggressively. Post-resection staging positron emission tomography-computed tomography (PET-CT) showed hypermetabolic mixed lytic and sclerotic lesion of T10 concerning for metastatic disease.

She received five cycles of adriamycin and ifosfamide chemotherapy. Adriamycin was discontinued due to left ventricular dysfunction with an ejection fraction of 30%-35%, multiple segmental abnormalities, diffuse left ventricular hypokinesis, and moderate to severe mitral valve regurgitation.

**Figure 2:**
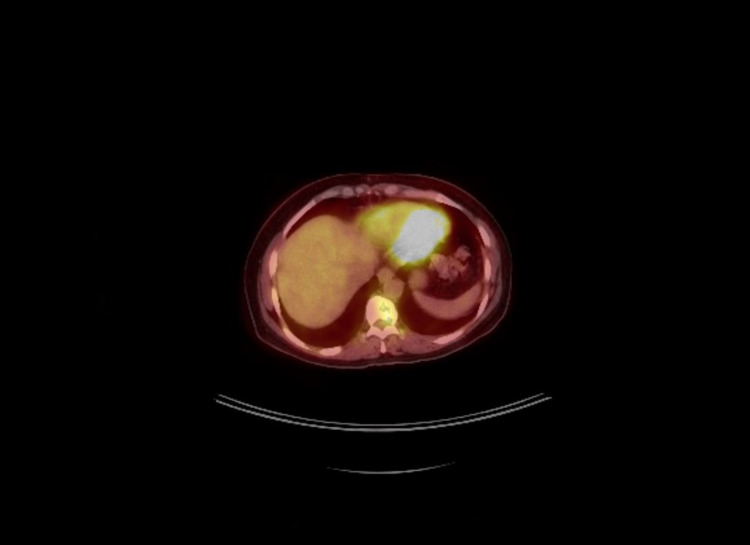
Post adjuvant therapy PET-CT with the hyper-metabolic T10 vertebra lesion

Despite intracardiac tumor, resection, concern for metastatic disease, chemotherapy, and systolic dysfunction, the patient was asymptomatic and had a robust performance status. A follow-up PET-CT five months after cessation of treatment did not show any evidence of new metastatic disease other than abnormalities in the T10 vertebra (Figure 2).

**Figure 3:**
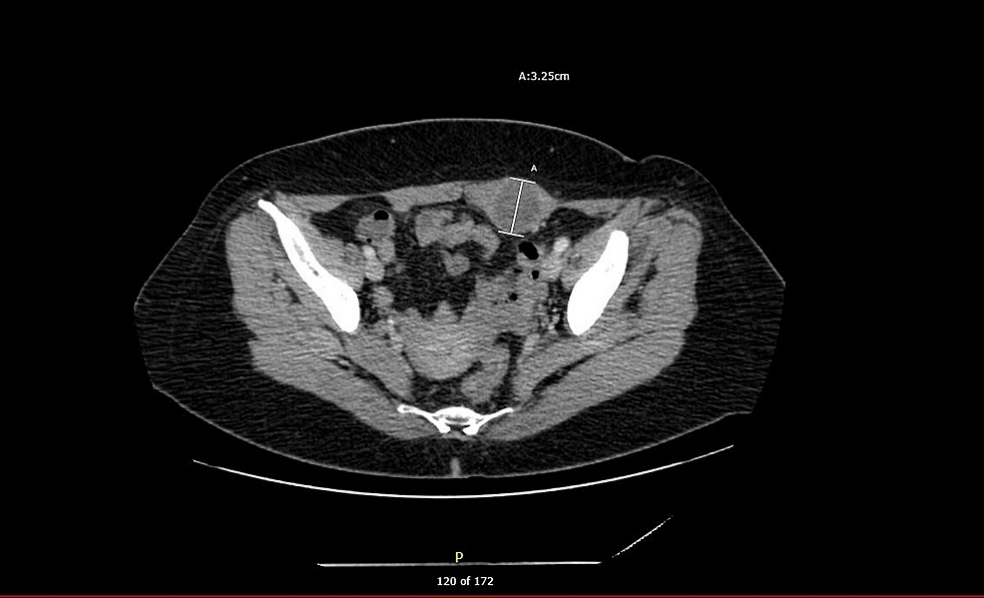
CT abdomen/pelvis with contrast for surveillance showing a 3.25 cm lesion in the left rectus abdominis

A repeat echocardiogram continued to reveal a depressed ejection fraction of 35%. A surveillance CT nine months after cessation of treatment revealed a 3.25 cm mass over the left rectus abdominis, a 2.2 cm subcutaneous lesion around T12, and a 4.4 cm mass medially in the left adductor muscle (Figure 3).

**Figure 4:**
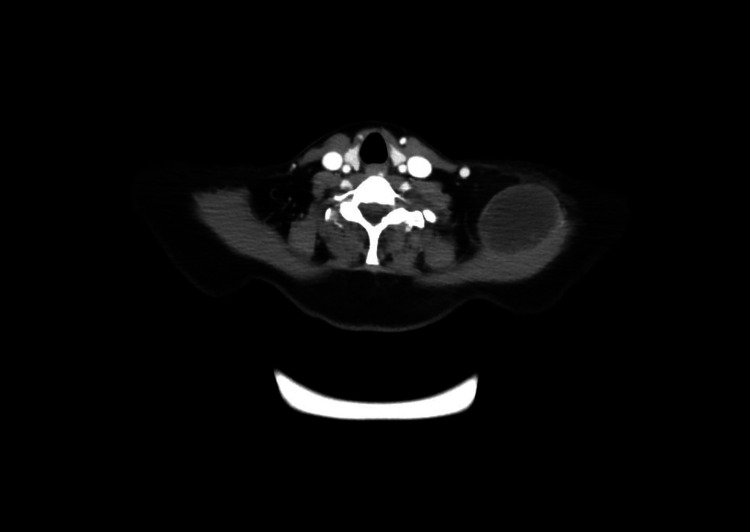
CT of chest/abdomen/pelvis showing progressive disease with a 4.4 x 5.5 cm left trapezius mass

Biopsy of these lesions confirmed high-grade osteosarcoma. She received gemcitabine and docetaxel as second-line therapy. Restaging scans after three cycles revealed explosive progression of the disease (Figure 4).

**Figure 5:**
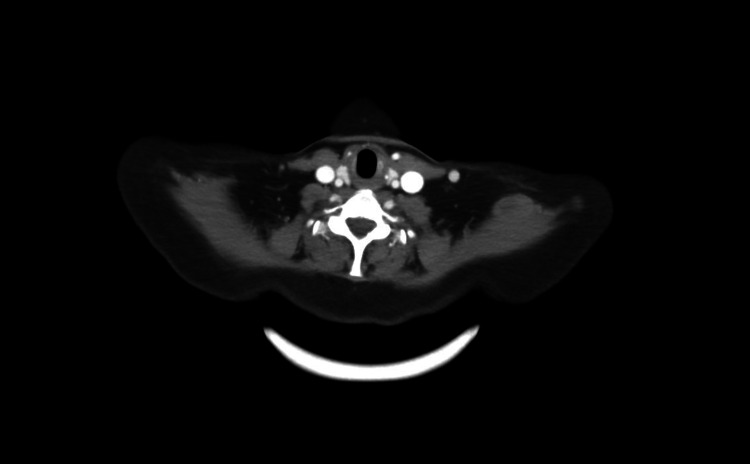
CT of chest/abdomen/pelvis with contrast showing disease response with the index lesion at the left trapezius decreased in size to 3.3 x 1.9 cms

She subsequently initiated cyclophosphamide and topotecan as third-line therapy and has had a dramatic response clinically and radiographically (Figure 5).

## Discussion

**Figure 6:**
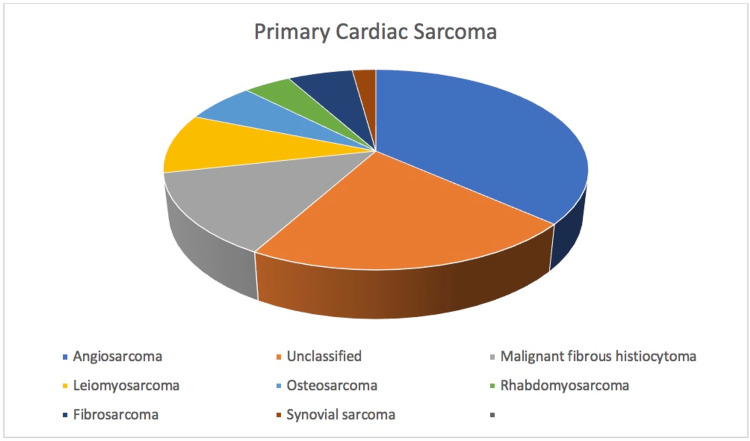
Incidence of primary cardiac sarcoma

Osteosarcoma is one of the most common primary skeletal tumors. Extra-skeletal osteosarcoma is relatively rare and is more often seen in lower extremity soft tissue. Cardiac osteosarcomas are extremely rare; they account for only 3%-9% of all cardiac sarcomas with the first case report in 1957 [5,6] (Figure 6). Fewer than 60 cases of primary cardiac osteosarcoma have been reported to date [6].

Primary cardiac tumors present with a wide variety of symptoms and signs masquerading as cardiovascular disease, usually as a combination of syncope, heart failure, arrhythmia, or embolic event [7]. The most common presenting symptom is shortness of breath. Intracavitary tumors are more likely to cause heart failure as opposed to intramural tumors that have a predilection for arrhythmias. Embolization of tumor pieces and local invasion of vital structures can also cause symptoms. Mass effect, obstruction, or functional effects cause a wide spectrum of signs and symptoms in malignant cardiac tumors. These include neurological deficits, palpitations, cough, hemoptysis, pleural effusion, chest pain, syncope, ankle edema, elevated liver enzymes (in the presence of right heart failure), and other symptoms of heart failure, valve dysfunction, and thrombus/tumor embolization [7,8]. 

Echocardiography is usually the initial imaging study performed to diagnose a cardiac tumor, however, CT and cardiac MRI offer distinct diagnostic advantages [9]. Echocardiography can evaluate the involvement of valves, size, and extension of the tumor. Benign tumors such as myxoma are pedunculated and usually on the left side whereas malignant tumors are often broad-based. Use of echocardiography contrast can help demonstrate the highly vascular nature of malignant tumors. A transesophageal echocardiogram is superior in diagnosing cardiac tumors located in the atrium. Echocardiography is usually followed by a CT or MRI which often provides a high degree of tissue characterization as well as assessment of calcification which can further aid in the diagnosis of these tumors. Cardiac osteosarcomas have been reported on cardiac CT as a low attenuation mass with dense calcification [9,10] (Table 1). 

**Table 1:** Primary cardiac malignant tumor characteristics

	Mean age at diagnosis in years	Frequency in %	Typical cardiac location	Gender predilection
Angiosarcoma	44	36	Right heart	No gender predilection
Malignant Fibrous histiocytoma	45	12	Left atrium	No gender predilection
Rhabdomyosarcoma	20	5	No particular preference	No gender predilection
Osteosarcoma	38	6	Left Atrium	Female predominance
Lymphoma	67	1	Right atrium	Male predominance

The treatment of primary cardiac tumors depends on location, size, histology, and metastatic spread. For treatment purposes, cardiac sarcomas are divided based on location into the right heart, left heart, and pulmonary artery sarcomas [11]. The main modality of treatment for primary cardiac tumors is surgical resection. Benign cardiac tumors such as myxomas are usually treated with surgical excision followed by clinical surveillance with imaging studies. Cardiac sarcomas have a poor prognosis as reported in many case studies, with a median survival of 6-25 months [10-12]. Radiotherapy is avoided in left and right heart tumors given the risk of myocardial toxicity, however, it can be used in the treatment of pulmonary artery sarcoma [11]. Given the rarity of cardiac sarcomas, there is a paucity of prospective randomized data on the efficacy of adjuvant chemotherapy or radiotherapy in addition to surgery. Based on limited evidence, multi-agent chemotherapy appears to be more effective, with doxorubicin, cisplatin, ifosfamide, taxanes, and gemcitabine having activity. A single-institution study done at a tertiary care center showed the most common first-line chemotherapy regimen included doxorubicin at 75 mg/meter sq given over 72 hours as a continuous infusion in combination with ifosfamide 10 g/meter sq divided over 4-5 days whereas gemcitabine 675-900 mg/meter sq on day 1 and day 8 with docetaxel 60-100 mg/meter sq on day 8 was the second most common regimen [8]. There is no evidence available on the use of neoadjuvant chemotherapy although theoretically it would be advantageous in obtaining clear surgical margins and help determine tumor response to therapy. This approach will need to be balanced with the urgency of the clinical presentation as in our case.

## Conclusions

Primary cardiac tumors are highly variable in presentation with non-specific symptoms, and have a poor overall prognosis. Given its rare nature, there is insufficient data to guide its management. Surgery remains the cornerstone in the treatment of cardiac sarcoma whereas the role of neoadjuvant and adjuvant chemotherapy requires further investigation.
